# Editorial: Neurodevelopment: parental influences, *in utero* exposures, and genetics, volume II

**DOI:** 10.3389/fnins.2026.1843049

**Published:** 2026-04-15

**Authors:** Kommu Naga Mohan

**Affiliations:** Molecular Biology and Genetics Laboratory, Department of Biological Sciences, Birla Institute of Technology and Science (BITS) Pilani Hyderabad Campus, Hyderabad, India

**Keywords:** copy number variants, mutations, neurodevelopmental disorders, opioid exposure, oxytocin

Neurodevelopmental disorders (NDDs) like autism spectrum, attention deficit hyperactivity, intellectual disability, motor dysfunction, and defects in communication show an incidence of 7–10% (Global Research on Developmental Disabilities Collaborators, 2018). Certain mental health disorders, that include defective brain development/maturation (schizophrenia, epilepsy, bipolar, etc.) display neurodevelopmental defects are also considered as NDDs (Owen et al., 2011; Colic et al., 2024; Bozzi et al., 2012). Together, these mental health disorders constitute another ~3% (Singh et al., 2023). Multiple causative factors such as genetic risk, fetal (intrauterine) environment (e.g., TORCH infections), substance abuse by the mother, nutritional deficiencies during pregnancy, perinatal risk factors (e.g., birth complications), exposure to certain environmental pollutants (e.g., lead) and post-natal factors such as trauma, infections in early childhood, extreme deprivation, neglect and parental stress (particularly maternal stress during pregnancy) have been suggested to play a role. Since these factors often can occur in combinations, a two-hit hypothesis was developed. For example, in schizophrenia the genetic risk can be the first hit and an environmental stressor in early or later life is the second (Bayer et al., 1999; Maynard et al., 2001). This is different from the classical two-hit hypothesis involving only genetic risk. For example, combined inheritance of both maternal (*CNTNAP2*) and paternal (*LRR4C*) deletions in an epileptic patient whose parents are normal (Servetti et al., 2021). Of note, many NDDs show higher prevalence rate in males than females, suggesting the influences of gender-specific physiology.

Despite vast information, much is left to be known on the nature, diversity and the combinations of the different categories of risk factors in NDDs. The present collection of articles is an attempt to address this gap and contains six reports ranging from the roles genetic risks to the gender-specific influences.

Rett syndrome (1 in 14,000 females) involves the X-linked *MECP2* mutations with a range of phenotypes (normal/mild to severe). These mutations are lethal in males. Cornelia de Lange syndrome also occurs at a similar frequency with no sex bias and presents with either severe (type 1 involving *NIPBL* mutations) or mild/moderate forms (type 2 and type 3 with mutations in the X-linked *SMC1A* and the autosomal *SMC3* genes, respectively). Here, Wang et al. report two cases of which one is a 7-year girl carrying mutations in both the genes showing delayed motor development, microcephaly, high-arched and thick eyes, intellectual disability, etc. The second one, is a 15-year male with staggering gait, tremors of extremities, high arched thick eyebrows, long eyelashes, delayed language development, microcephaly, etc. with an inherited *PMP22* duplication (associated with Charcot-Mary-Tooth disease) and a *de novo* missense mutation in *SMC3*. Both dual-positive genetic variants involved blended or novel phenotypes. Buratti-Harel syndrome, a rare disorder with 16 cases reported so far is due to *SIAH* mutations. Zheng et al., as part of this Research Topic, describe a case with a predicted to destabilizing missense mutation in the Zinc finger 1 domain of SIAH. Based on phenotypic analysis of other mutations, the authors suggest broad phenotypic heterogeneity when mutations occur in the Zinc finger domains but severe developmental delay and reproductive abnormalities if they are in the RING finger domain. However, this phenotypic bifurcation needs confirmation through analysis of more patients and development of animal models. Analysis of *ZIMZ1* mutations constitute the third example of rare mutations associated with NDDs. To better understand the variable phenotypes, Cormier et al. used the mutation data available on 35 individuals and suggest that mutations in the TPR (tetratricopeptide repeat) domain are more likely associated with intellectual disability, motor delay and other physical manifestations (facial dysmorphism, distal skeletal abnormalities, etc.). On the other hand, mutations proline-rich domains seem to be associated with autism spectrum disorder co-occurring with intellectual disability. *SCN2* mutation analysis constitutes the fourth manuscript focusing on NDD-associated mutations. *SCN2* mutations are associated with difficult to treat seizures, severe developmental delays, autism spectrum disorder, etc., but the information on prenatal phenotypes is limited. In two prior reports, *de novo* mutations in *SCN2* showed ventriculomegaly, central nervous system dysplasia, poor distinction between cortical and subcortical layers, abnormal neural migration and corpus callosum dysgenesis at 30th and 32nd gestational weeks (Sauvestre et al., 2017; Bernardo et al., 2017). Post-natally, there was focus status epilepticus on day 6 (Bernardo et al., 2017). In this collection of articles, Zhu et al. describe the third report on prenatal phenotypes of an embryo with a *de novo* frameshift mutation. At 21st gestational week, the fetus showed a cavum septum pelucidi (fluid-filled cavity between the thin leaflets of the septum pellucidum, a thin partition separating the anterior horns of the left and right ventricles). At 24th and 28th weeks, there was progressive widening of this cavity, confirming cortical thinning and ventriculomegaly as the associated phenotypes.

As mentioned above, distorted male-female ratios are observed in multiple NDDs of which, autism spectrum disorder is one of the prominent with a ratio of 3:1 (Loomes et al., 2017). Investigations of gender-specific physiological factors can help explain this difference. Using a prenatal valproic acid exposure rat model, Wen et al. investigated the effects of oxytocin administration on male autistic rats and showed ameliorative effects on the observed behavioral abnormalities. Valproic acid specifically affected neurodevelopment-associated genes with a significant suppression of those involved in oligodendrocyte development and differentiation associated with the PI3K/AKT pathway. These defects were relieved by oxytocin. These results, viewed in the context of lower oxytocin levels reported in autism spectrum patients (John and Jaeggi, 2021), its higher levels in females provides evidence in support of the gender-specific difference. However, increased oxytocin levels in males may result in hypersexual behavior (Flanagan et al., 2022).

The sixth manuscript in this Research Topic deals with the comparative metabolite analysis in the dorsal anterior cingulate cortex of pregnant women treated with medications for opioid use disorder (MOUD) with and without polysubstance use. By employing 1H-MR spectroscopy, Class et al. observed a positive correlation with elevated choline-to-creatine ratio in MOUD with polysubstance exposure. This increased ratio is also reported in ADHD, schizophrenia and bipolar disorders (e.g., Jin et al., 2001; Galińska-Skok et al., 2018). But the results are not consistent (e.g., Valizadeh et al., 2025). Clearly more data is needed to understand the relationship between the choline-to-creatine ratios and these disorders.

Together, this collection of articles/case reports extend the influences of parental/*in utero* exposures (e.g., prenatal MOUD exposure) and genetic risk on neurodevelopmental process and the associated disorders.
